# Organisational and Governance Conditions Shaping Psychological Safety and Structural Vulnerability in Float Pool Nursing: A Qualitative Study

**DOI:** 10.1155/jonm/1427120

**Published:** 2026-07-19

**Authors:** Rubén Llada Suarez, Marta Manuz Pérez, Yolanda García Rodríguez, Margarita González Pérez, Carmen Álvarez Fanjul, Maria Antuna-Casal, Lucía del Fresno Marqués

**Affiliations:** ^1^ Department of Medicine, Nursing Area, University of Oviedo, Oviedo, Spain, uniovi.es; ^2^ Nursing Coordination Unit, Hospital Universitario Central de Asturias (HUCA), Oviedo, Spain, hca.es

**Keywords:** flexible staffing, float pool nursing, organisational culture, psychological safety, transformational leadership, workforce sustainability

## Abstract

**Background:**

Healthcare organisations increasingly rely on flexible staffing models to maintain continuity and quality of care amid demand volatility, workforce shortages and rising clinical acuity. Float pool staffing is central to these models, yet the organisational and leadership conditions required for safe mobile nursing practice remain insufficiently understood.

**Aims:**

To explore the lived experience of registered nurses and nursing assistants working within a hospital float pool and examine what their accounts reveal about the organisational and leadership conditions required for sustainable mobile workforce models.

**Methods:**

A descriptive phenomenological study, informed by Husserlian philosophy and Colaizzi’s method, was conducted in a large tertiary hospital in Spain. Semistructured interviews were undertaken between March and May 2025 with a purposively selected sample of float pool staff (*n* = 12; six registered nurses and six nursing assistants). A brief preparatory contextual checklist informed interview guide refinement and contextual understanding without constituting a separate quantitative analytic component. Data were analysed through iterative immersion, coding and thematic development. Reporting followed COREQ and SRQR guidance.

**Results:**

Float pool practice emerged as structurally vulnerable work shaped by uncertainty, inconsistent expectations and fluctuating legitimacy across units. Mobility disrupted psychological safety, belonging, and the enactment of expertise, requiring continuous adaptation and emotional regulation. Inadequate induction, blurred role boundaries and uneven supervisory support intensified vulnerability, whereas inclusive and supportive leadership behaviours were experienced as protective.

**Conclusions:**

Sustainable mobile staffing is not secured by individual adaptability alone. It depends on organisational governance that reduces uncertainty, standardises induction and role clarity and embeds supportive leadership and reflective structures across receiving units. Float pool practice, therefore, requires organisational rather than purely individual solutions.

**Implications for Nursing Management:**

Nursing managers should recognise float pool nursing as a specialised area of practice requiring defined competencies, consistent orientation and leadership practices that foster psychological safety, inclusion and workforce sustainability across mobile staffing systems.

**Key Points for Nursing Managers**
• Recognise float pool staff as a structurally vulnerable workforce whose psychological safety, role legitimacy and capacity for effective practice depend on organisational conditions, not individual adaptability alone.
• Ensure that flexible staffing models are underpinned by explicit role expectations, consistent induction and culturally coherent reception practices across receiving units.
• Strengthen leadership practices that foster inclusion, support and relational continuity, as these operate as protective conditions for mobile staff across variable clinical environments.
• Reduce operational uncertainty by standardising early‐shift orientation, clarifying delegation boundaries and strengthening unit‐level communication with float pool staff.
• Provide protected access to reflective and supportive spaces that sustain well‐being, learning and retention within mobile workforce models.
• Position float pool nursing as a specialised area of practice requiring defined competencies, organisational recognition and sustained managerial investment.

## 1. Introduction

Healthcare systems worldwide are facing escalating organisational pressures driven by workforce shortages, increasing patient acuity, fluctuating service demand and persistent instability in staffing structures. Within this landscape, flexible staffing models have become essential for maintaining continuity, safety and quality of care. In this study, float pool nursing refers to a hospital staffing model in which registered nurses and nursing assistants are redeployed across multiple units according to operational need rather than being attached to a single ward. We use mobile nursing to refer more broadly to forms of nursing work characterised by cross‐unit movement, shifting clinical environments and variable team belonging. Workforce agility, understood here as the organisational capacity to redeploy nursing staff efficiently across units and clinical contexts in response to changing demands, has, therefore, emerged as a strategic requirement for operational resilience in environments characterised by volatility and resource constraint [1, 2]. Although agility enhances organisational responsiveness, it simultaneously reshapes the conditions under which registered nurses work, introducing demands related to uncertainty, rapid adaptation and relational discontinuity that remain insufficiently understood from a managerial perspective.

Growing evidence suggests that these staffing models are embedded within broader patterns of structural vulnerability. Structural vulnerability refers to the ways in which organisational arrangements, inequities in resources and systemic constraints generate differential exposure to risk, stress or marginalisation among healthcare professionals [3]. Mobile staffing roles are particularly susceptible to these dynamics, as they often confront uneven access to orientation, fragmented communication channels, variable support in receiving units and fluctuating expectations regarding their competence and responsibilities. Classic organisational ethnographies have demonstrated that nursing work becomes invisible when institutional routines conceal the cognitive labour, anticipatory judgement and adaptive expertise required for safe practice [4]. These invisibilities are intensified in roles that lack stable relational belonging, contributing to emotional strain, uncertainty and a diminished sense of professional legitimacy.

Parallel to these concerns, the concept of organisational resilience has gained prominence as healthcare systems seek to sustain performance under conditions of chronic pressure. Registered nurses are increasingly recognised as central agents of system resilience, particularly during periods of disruption or high operational demand [5]. Contemporary research conceptualises resilience as a multilevel construct shaped not only by individual coping capacities but also by leadership practices, relational climates, resource adequacy and organisational learning processes [6, 7]. Higher resilience is associated with stronger work engagement, lower turnover intention and improved quality of care [8]. Yet excessively relying on resilience as a compensatory expectation can impose unintended burdens; scholars warn against overemphasising resilience without addressing structural conditions that generate stress, framing this dynamic as the ‘dark side of resilience’ [9].

In parallel, *transformational leadership* (TL) has developed into a central theoretical and empirical pillar within nursing management. Transformational leaders articulate a shared vision, foster psychological safety, stimulate professional growth and provide individualised support. Recent studies show that TL improves nursing care performance, care quality and patient outcomes [10, 11], enhances leadership competencies among nurse managers [12], promotes sustainability and organisational achievement [13] and contributes to healthier work environments [14]. TL has also been associated with increased nurse well‐being, mediated through organisational justice and quality of work life [15], and with stronger work engagement and professional fulfilment [16]. Additional work demonstrates its influence on safety practices [17], nurse retention [18] and strategic flexibility, supporting competence development in dynamic clinical settings [19]. Taken together, these findings position TL as a protective organisational structure capable of buffering uncertainty and strengthening workforce sustainability.

Despite the robustness of this evidence, a significant gap persists at the intersection of leadership, structural arrangements and workforce mobility. Most leadership studies focus on nurses embedded in stable ward‐based teams, where relational continuity, team identity and role clarity differ markedly from the realities faced by mobile staff. The limited empirical research on floating or mobile nursing roles suggests heightened stress, marginalisation, inconsistent expectations and emotional dislocation [20–22]. Recent studies reveal that float pool registered nurses encounter distinctive job demands and resource constraints, often linked to limited preparation, uneven reception in temporary units and fragmented supervisory structures [23]. These challenges intersect with factors such as autonomy, resilience and fatigue, which can shape turnover intentions and missed care [24, 25]. Early‐career registered nurses further describe mobility and rapid learning demands as both growth opportunities and sources of vulnerability when adequate support is lacking [26]. Collectively, this literature indicates that mobility‐based staffing roles embody both organisational potential and relational fragility yet remain underexamined in contemporary management research.

As hospitals increasingly rely on flexible staffing systems to stabilise operations amidst workforce instability, understanding the lived experience of mobile nursing professionals becomes essential for advancing effective leadership strategies and organisational policies. Yet, the literature still offers limited understanding of the leadership, organisational and governance conditions that shape psychological safety, role legitimacy and sustainable practice in mobile nursing roles. While supportive structures such as clinical supervision have been associated with improved well‐being and reflective capacity, their integration within mobile staffing systems remains conceptually and practically underdeveloped [27]. Examining how mobile registered nurses interpret their work, navigate uncertainty and articulate their support needs can, therefore, reveal critical insights into the organisational conditions that enable or constrain effective performance.

This qualitative study explores the lived experience of registered nurses and nursing assistants working within a mobile staffing model in a large tertiary hospital. Specifically, the study was guided by the following research question: How do float pool registered nurses and nursing assistants experience the organisational, relational and leadership conditions that shape psychological safety, structural vulnerability and sustainable mobile nursing practice? By analysing how these professionals describe their roles, the tensions they encounter and the forms of support they perceive as necessary, the study aims to inform evidence‐based leadership and management strategies that strengthen flexible staffing systems and enhance workforce sustainability.

## 2. Methods

### 2.1. Study Design

The study employed a qualitative design grounded in descriptive phenomenology, drawing on Husserl’s philosophical foundations [28] and Colaizzi’s methodological adaptation for descriptive phenomenological analysis [29]. This design was selected to examine, with descriptive fidelity, the lived experience of float pool registered nurses and nursing assistants working under conditions of high mobility, cross‐unit cultural variability and fluctuating clinical demands. The phenomenological orientation guided both data generation and interpretation by prioritising participants’ first‐person accounts and the articulation of essential meanings that organise everyday practice.

To enhance transparency and reporting completeness, reporting was guided by SRQR; for the interview component, we used the COREQ checklist and provided it as Supporting File S2 [30, 31]. Prior to the qualitative interviews, a brief author‐developed fourteen‐item contextual checklist was completed by the full float pool workforce during the study period (*N* = 22). The checklist was generated by the research team as a preparatory contextual mapping tool, informed by the study aim, the organisational characteristics of float pool work, and the interview areas intended for exploration in the interview guide. It focused on perceived workload intensity, stress exposure, perceived competence adequacy, vulnerability experiences and training or supervision needs within the float pool team.

Contextual checklist data were used solely as an orienting and sensitising input to refine the interview guide and enhance contextual attunement to potentially salient concerns. They did not constitute a separate quantitative analytic component, were not intended as a validated measurement instrument and did not substitute for the interpretive work of the phenomenological analysis. Following this preparatory phase, twelve key informants who met the inclusion and exclusion criteria were purposively selected for the phenomenological interview phase, with recruitment continuing until data saturation was reached. Descriptive outputs from this preparatory phase are presented in Supporting File S6. Leadership is examined through participants’ perceptions of organisational and supervisory practices within receiving units and the float pool structure. TL is, therefore, employed as a sensitising interpretive lens rather than as a quantitatively measured construct.

### 2.2. Setting

The study was conducted in a large tertiary hospital in Spain (Western Europe, Atlantic Coast) that operates a float pool team responsible for providing flexible, cross‐unit staffing cover in response to fluctuating service demand and staffing instability. The inherent mobility of this role, together with variability in unit cultures and the unpredictability of clinical workloads, provided a meaningful organisational context in which to explore how professional experience and perceived safety are constituted within a model characterised by uncertainty, rapid adaptation and ongoing organisational change. To minimise the risk of deductive disclosure in a small and identifiable workforce, the hospital is described but not named, and locality‐specific or uniquely identifying organisational details are not reported.

### 2.3. Sampling and Participants

Purposive sampling was used to recruit participants from the hospital float pool team in order to obtain information‐rich accounts from staff with sufficient experiential breadth to reflect on cross‐unit practice. The source population comprised the hospital float pool workforce during the study period (*N* = 22), consisting of eleven registered nurses and eleven nursing assistants assigned to the 24‐h on‐call coordination structure and deployed to reinforce clinical units according to occupancy, workload or staffing shortages. All members of this workforce completed the preparatory contextual checklist.

From this wider workforce, semistructured interviews were conducted with a purposively selected sample of key participants who met the inclusion and exclusion criteria. Data saturation was reached with twelve informants: six registered nurses (RN1–RN6) and six nursing assistants (NA1–NA6).

Inclusion criteria were active participation in the float pool model, stability in the role, sufficient tenure to speak reflectively about mobile practice and willingness to participate. Individuals who were newly incorporated into the role, were not actively working within the float pool team during the study period or did not wish to participate were not included.

The final sample consisted of twelve professionals, including six registered nurses and six nursing assistants. Participants were approached through the float pool coordination structure and received verbal and written information about the study, and those willing to participate provided written informed consent. This purposive sample was considered appropriate to capture a sufficiently information‐rich and professionally varied cross‐section of the float pool team. Recruitment continued until meaning‐focused saturation was achieved, understood as the point at which additional interviews no longer yielded substantively new insights or meaningful variations in the developing analytic account [32]. Participant characteristics from the contextual checklist are summarised in Supporting Table S6_1.

### 2.4. Preparatory Procedures

Prior to data collection, all study materials were developed, including participant information sheets, written consent forms and a detailed protocol outlining the methodological sequence and safeguards for confidentiality. Ethical approval was obtained from the Ethics Committee for Research with Medicines of the Principality of Asturias (CEImPA 2025.082), and institutional authorisation was granted by hospital leadership. These preparatory steps ensured compliance with ethical and organisational standards for participant protection and scientific integrity.

### 2.5. Data Collection

**FIGURE 1:**
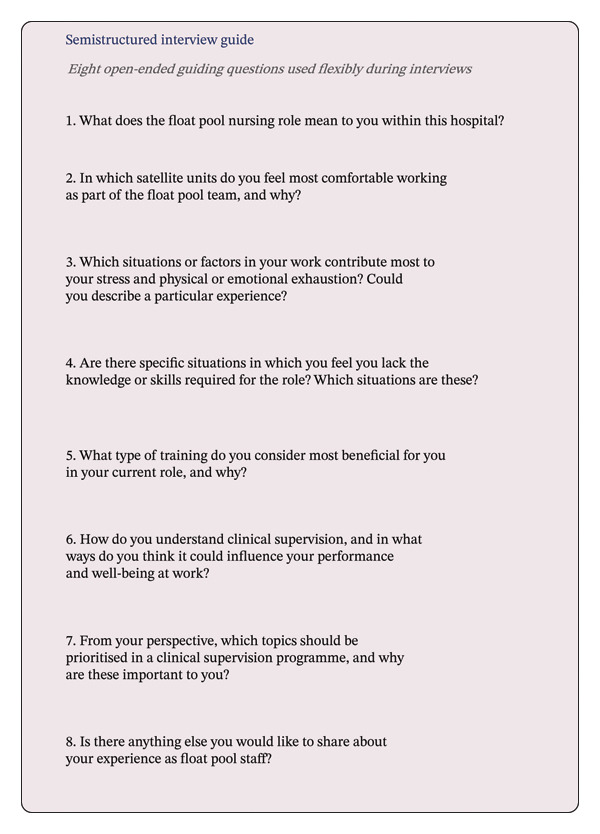
Semistructured interview guide used for data collection. Note. The interview guide comprised eight open‐ended guiding questions used flexibly during the interviews. These questions were intended to support conversational coverage of role meaning, adaptation across units, stressors, perceived competence, training needs, clinical supervision and additional experiences. They were not treated as prespecified analytic categories. The qualitative findings are reported separately as themes generated through the phenomenological analysis.

Data were generated through individual semistructured interviews conducted in a quiet, private room within the hospital setting. The interview format was designed to elicit detailed narratives of working across units, including experiences of adaptation, perceived support, relational and cultural discontinuities, and organisational conditions shaping safe practice. Interviews were guided by an eight‐question interview schedule exploring role meaning, comfort across units, stressors, perceived competence, training needs and perceptions of clinical supervision (Figure 1; Supporting File S1).

Interviews lasted approximately 30–60 min, were audio recorded with participant consent, transcribed verbatim and anonymised to preserve confidentiality. No follow‐up interviews were conducted. Interviews were conducted in Spanish. Quotations presented in English in the manuscript were translated by the authors for reporting purposes while preserving semantic fidelity to the original accounts. Field notes were taken during and immediately after each interview to capture contextual information, pauses, interruptions, nonverbal cues, and the emotional tone of interactions; these notes informed analytic reflexivity but were not treated as standalone data. Data collection took place between March and May 2025. Additional reporting detail is provided in Supporting File S2.

### 2.6. Analytical Procedures

**FIGURE 2:**
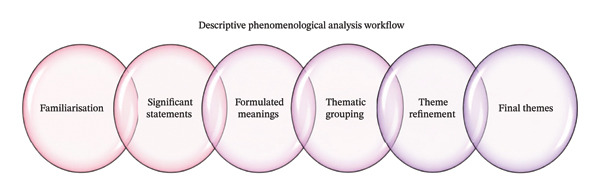
Analytic workflow used for descriptive phenomenological analysis. Note. The figure provides a simplified visual representation of the iterative analytic process used to organise participants’ accounts into phenomenological themes. It is intended to support transparency of the analytic workflow and should be read alongside the detailed description of Colaizzi’s method in the text.

Data analysis followed Colaizzi’s descriptive phenomenological method [29], maintaining a commitment to descriptive attentiveness to participants’ accounts while progressively clarifying shared meanings. The first analytic phase involved immersive familiarisation with the dataset through repeated, reflective reading of all transcripts to apprehend narrative flow, emotional tone and contextual texture. The analytic workflow applied in this study is summarised in Figure 2.

Following familiarisation, significant statements relevant to the experience of float pool work were identified and extracted across transcripts. These statements were then examined to formulate meanings that remained grounded in participants’ descriptions while allowing analytic articulation beyond isolated quotations. Formulated meanings were compared across participants and clustered into thematic groupings that captured patterned experiential structures and meaningful variation.

To enhance procedural clarity in the transition from transcript fragments to thematic families, selected phases of Braun and Clarke’s thematic analysis [33, 34] were used pragmatically as a coding and theme‐development pathway within the broader phenomenological logic of Colaizzi’s method. In practice, this involved (1) repeated reading and familiarisation; (2) identification of significant statements and meaning units; (3) formulation of meanings; (4) clustering of meanings into candidate thematic groupings; (5) iterative review and refinement of those groupings and (6) definition and naming of the final experiential themes.

Thus, Braun and Clarke’s phases supported analytic organisation, whereas Colaizzi’s framework remained the primary phenomenological orientation guiding interpretation and descriptive fidelity.

Analysis was supported by OpenCode 4.3 (Umeå University), which was used as an organisational and audit‐support tool to manage quotations, assign and refine codes, cluster coded segments into thematic families, and document analytic memos and interpretive decisions over time. Disagreements in coding or thematic interpretation were addressed through structured analytic discussion among members of the research team until consensus was reached.

**FIGURE 3:**
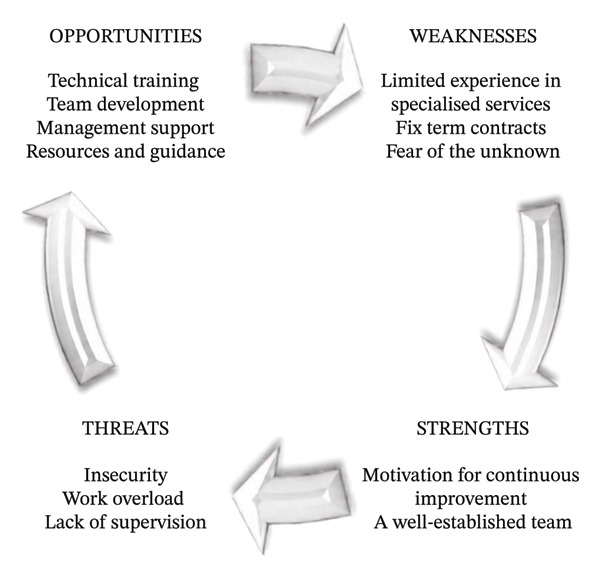
SWOT analysis of the float pool nursing role. Note: The SWOT framework was used as an analytic device to synthesise key experiential dimensions identified in the qualitative data. Categories reflect participants’ accounts of organisational conditions shaping float pool practice rather than formal organisational evaluation and are presented to support interpretive clarity.

To support managerial interpretation and practical application, the final themes were synthesised into a SWOT matrix presented in Figure 3, translating experiential findings into organisational strengths, vulnerabilities, opportunities and threats relevant to safe practice within float pool staffing models. Supporting Files S3–S5 provide additional traceability regarding coding, audit trail decisions and management translation.

### 2.7. Trustworthiness

Trustworthiness was ensured through strategies aligned with the criteria of credibility, dependability, confirmability and transferability articulated by Lincoln and Guba [35]. Credibility was strengthened through investigator triangulation and structured analytic discussions in which codes and candidate thematic groupings were reviewed, challenged and refined, alongside continuous comparison between transcript excerpts and emerging interpretations to preserve descriptive fidelity. Dependability was supported by maintaining a detailed audit trail documenting methodological steps, coding decisions, theme development and successive refinements to the analytic structure. Confirmability was enhanced through the systematic use of analytic memos and a reflexive journal, which captured assumptions, interpretive decisions and the rationale for analytic choices, thereby supporting transparency and traceability of interpretations back to the data [35]. Transferability was addressed through thick description of the organisational context and of the narrated work conditions, enabling readers to judge the applicability of findings to comparable settings [35]. Interview transcripts were not returned to participants for comment or correction. This decision was taken to minimise participant burden and to reduce the risk of reidentification within a small and highly recognisable professional group, while remaining consistent with the descriptive phenomenological focus of the study. Additional reporting detail regarding rigour procedures is provided in Supporting File S2.

### 2.8. Researcher Positioning

Data collection was undertaken with two researchers present. One researcher led the semistructured interview, while the second managed audio recording, took field notes and observed relevant nonverbal and contextual cues. One researcher was linked to the hospital on‐call coordination structure and, therefore, had organisational proximity to the float pool context; the second was external to the float pool structure and affiliated with the research and education department. This combination supported contextual understanding while preserving a degree of analytic distance. The researcher linked to the coordination structure had organisational proximity rather than a formal employment, disciplinary or performance‐evaluative role over participants within the research process. Nevertheless, because such proximity could potentially influence participation or disclosure, it was explicitly managed through ethical and reflexive safeguards. Participants received written and verbal information emphasising voluntary participation and the possibility of withdrawal without consequences. Interviews followed a preagreed semistructured interview roadmap, and data were anonymised before wider analytic discussion. Analytic credibility and reflexive control were further supported through investigator triangulation, blinded review of anonymised materials where appropriate, field‐note comparison and audit‐trail documentation of analytic decisions. Reflexivity was treated not as a disclosure statement but as an ongoing methodological practice of ‘negotiating the swamp’ of research relations, interpretive commitments, and potential blind spots, consistent with Finlay’s account of reflexivity as both opportunity and methodological challenge [36].

Analytic memos and reflexive journaling were used to make assumptions, positional influences, and interpretive decisions visible and reviewable across the study process. This reflexive stance contributed to transparency and methodological strength by making positional influences visible and analytically accountable, in line with established recommendations for reflexivity in qualitative inquiry [36]. Additional reporting detail is provided in Supporting File S2.

Given the research team’s contextual familiarity with the float pool setting, potential preconceptions were considered during the development of the contextual checklist, the interview guide and the management translation matrix. Assumptions concerning mobility, workload, stress, preparedness, supervision and role recognition were discussed within the research team and treated as sensitising concerns rather than predetermined analytic categories. The management translation matrix was developed after theme generation, and contextual checklist descriptors were used only to support practical prioritisation.

## 3. Results

### 3.1. Contextual Checklist: Descriptive Overview

A brief author‐developed contextual checklist was completed by the full float pool workforce during the study period (*N* = 22) to provide contextual orientation for the qualitative phase, without constituting a mixed‐methods design. These contextual data refer to the wider float pool team, whereas the phenomenological interviews were conducted with a purposively selected sample of twelve key informants. The team was highly experienced (75% reporting > 6 years in the float pool role) and characterised by high clinical mobility and largely unstructured prior mobility training. Descriptive responses indicated common stress and preparedness gaps, alongside limited awareness and uptake of clinical supervision. Characteristics of the wider float pool workforce are summarised in Supporting Table S6_1, and additional contextual checklist distributions are reported in Supporting File S6 (Figures S6_1–S6_2; Tables S6_1–S6_5).

These contextual data help situate the qualitative inquiry within the wider float pool workforce and provide descriptive background for interpreting the experiential findings reported below.

### 3.2. Qualitative Findings

Findings are reported as four qualitative themes generated through the phenomenological analysis. Figure 1 presents the semistructured interview guide used for data collection; it was not treated as a set of prespecified analytic categories.

To foreground role‐specific patterns within the shared experience of float pool work, Table 1 summarises cross‐cutting contrasts between registered nurses and nursing assistants. Results are grounded in participants’ descriptions of day‐to‐day work and are organised into experiential themes that capture the organisational conditions shaping mobility, uncertainty and identity.

**TABLE 1:** Role‐specific contrasts between registered nurses and nursing assistants organised by qualitative theme.

Theme	Common experiences	Nursing assistants: distinctive emphases	Registered nurses: distinctive emphases
Theme 1. Polyvalence as identity and structural demand	Both groups valued polyvalence, adaptability and the opportunity to support overloaded units, while also describing intermittent invisibility and uneven recognition.	Nursing assistants emphasised the need for clearer task boundaries and protection from inappropriate delegation or assumptions about their role.	Registered nurses described a stronger cognitive and emotional burden linked to expectations that they should function competently across diverse and sometimes specialised clinical areas.
Theme 2. Uncertainty, stress and formative vulnerability	Both groups described uncertainty on arrival, variability across units, lack of anticipatory information and stress linked to perceived competence gaps.	Nursing assistants stressed the importance of immediate practical instructions, predictable routines and clear unit‐specific guidance.	Registered nurses more often described stress in high‐acuity or specialised settings, concern about clinical risk and the need for structured rotations or refresher training to maintain confidence.
Theme 3. Clinical supervision as emotional and developmental scaffolding	Both groups understood supervision as support, guidance, reflection and professional development rather than control.	Nursing assistants emphasised supervision as a source of relational clarity, protection from unrealistic expectations and support in managing communication or conflict.	Registered nurses emphasised feedback, debriefing and a reference person who could support decision‐making and confidence in unfamiliar or complex clinical environments.
Theme 4. Belonging, recognition and the quest for role definition	Both groups linked psychological safety to welcome, recognition, inclusion and legitimate placement within receiving units.	Nursing assistants highlighted role definition and delegation safeguards as central to preventing conflict and preserving collaborative practice.	Registered nurses described threats to professional legitimacy when their expertise was questioned or when they were deployed without sufficient contextual preparation.

*Note:* The table is organised according to the four qualitative themes reported in the Results section. It summarises role‐specific emphases within each theme rather than presenting separate analytic categories.

Leadership appears in these findings as perceived supervisory and organisational practices, communication, availability, recognition, guidance and role clarification, rather than as a formally assessed construct. Accordingly, references to leadership in this section should be read as accounts of experienced support (or its absence) within units and across the float pool structure. TL is not operationalised or quantitatively measured in this study; it is employed as a sensitising interpretive lens to frame the discussion and to connect the qualitative patterns to established leadership theory.

### 3.3. Theme 1. Polyvalence as Identity and Structural Demand

This theme captures how float pool staff experienced polyvalence not merely as a functional requirement but as a professional identity shaped by constant redeployment, organisational usefulness and uneven recognition. Participants consistently described the float pool role as marked by high versatility, rapid adaptability and constant mobility. They portrayed themselves as professionals who ‘go where they are needed’ and ‘support overloaded teams’, assuming organisational responsibility beyond a fixed unit identity.

Polyvalence emerged not only as a skill set but as an identity. Registered nurses and nursing assistants positioned themselves as structural supports of the hospital, reinforcing continuity of care during peaks of demand or staffing gaps. Yet this same polyvalence created a dual burden: Recognition for being essential coexisted with invisibility when their contribution was taken for granted. This theme also suggests that organisational expectations of autonomous adaptability may become psychologically costly when they are not accompanied by recognition, guidance, or inclusive team cultures.

### 3.4. Theme 2. Uncertainty, Stress and Formative Vulnerability

This theme refers to the situational, relational and educational vulnerabilities produced when staff move across units without stable orientation, anticipatory information, or consistent support. Uncertainty constituted a foundational element of the float pool experience. Participants described stress arising not only from clinical overload but also from situational ambiguity: arriving in unfamiliar units, lacking orientation, entering emotionally distressed teams and navigating shifting expectations without prior briefing or support.

These situational stressors were consistent with the descriptive contextual checklist patterns, which were used only to contextualise the interview findings and were not treated as a separate quantitative analytic strand.

Emotional contagion, perceived judgement by receiving teams and role ambiguity further intensified this burden.

Educational gaps amplified this vulnerability. Registered nurses identified deficits in critical care, paediatrics, emergency services and the emergency gynaecology unit, while nursing assistants expressed insecurity in pharmacy, endoscopy, operating theatres and the central sterile supply department (CSSD). Participants frequently linked emotional strain with insufficient training, emphasising that the stressor was ‘not physical effort, but lack of specific skills’.

This theme situates formative vulnerability as a key leadership concern: In transformational models, training, guidance and psychological support are crucial levers for strengthening professional identity and mitigating stress. The absence of structured induction or ongoing learning opportunities impeded these protective mechanisms.

### 3.5. Theme 3. Clinical Supervision as Emotional and Developmental Scaffolding

This theme concerns participants’ understanding of supervision as a relational, reflective, and developmental structure capable of reducing uncertainty and strengthening competence, well‐being and belonging. When reflecting on clinical supervision, participants consistently expressed an understanding aligned with TL principles: supervision as relational support, professional development and emotional containment rather than control or correction.

Supervision was described as a source of security, clarity and guided reflection. Registered nurses valued constructive feedback, help in identifying strengths and weaknesses, and a reference figure who could accompany them through unfamiliar clinical territories. Nursing assistants emphasised empathy, accessibility and protection from unrealistic expectations.

The priority elements identified in Supporting Table S6_5—structured induction, unit‐specific protocols, conflict mediation, emotional support and reflective meetings—reveal a clear organisational demand for leadership practices grounded in presence, empathy and shared meaning‐making.

This theme demonstrates that participants not only desire supervision but also conceptualise it as a leadership encounter capable of transforming their sense of competence, well‐being and belonging.

### 3.6. Theme 4. Belonging, Recognition and the Quest for Role Definition

This theme captures how psychological safety was closely tied to experiences of welcome, recognition and legitimate role placement across receiving units. Belonging emerged as one of the strongest protective factors against stress and uncertainty.

Participants described the float pool team as a supportive community and expressed a deep appreciation for cohesive relationships and mutual care. Positive reception in receiving units markedly improved confidence, performance and well‐being.

Conversely, lack of clarity regarding assigned tasks, occasional underutilisation or misallocation of responsibilities, and insufficient recognition created emotional and professional dissonance. Nursing assistants reported that a clearer definition of role boundaries could prevent conflict and unrealistic expectations.

This theme underscores the relational dimension of leadership. TL emphasises inclusion, recognition and shared purpose, elements shown here to directly influence psychological safety, motivation and job satisfaction.

### 3.7. Cross‐Cutting Contrasts Between Registered Nurses and Nursing Assistants

A cross‐cutting analysis revealed substantial common ground between registered nurses and nursing assistants, alongside meaningful divergences that illuminate the nuanced dynamics of float pool practice.

These contrasts were not abstract category differences but were grounded in recurring role‐specific accounts in the interviews; as one nursing assistant explained, ‘When you arrive… no one tells you anything; you feel completely disoriented at first…’ (NA1).

Both groups valued polyvalence as a professional strength, viewing adaptability and broad clinical exposure as assets that enriched their competence. They also described uncertainty as a persistent source of stress, emphasised the need for structured, practice‐based training and expressed appreciation for the dynamism inherent in the float pool role.

Yet important differences emerged. Nursing assistants highlighted the necessity of clearly defined role boundaries to prevent inappropriate task allocation and to preserve clarity in collaborative practice. Registered nurses, by contrast, more frequently articulated emotional strain linked to the specialised clinical contexts into which they were deployed, noting the complexity and cognitive load associated with unfamiliar high‐acuity environments. They also expressed a stronger need for structured rotations to maintain competence and confidence when working across units with advanced clinical demands.

These contrasts underscore the importance of leadership adopting differentiated yet coherent approaches that address the specific needs, vulnerabilities and professional expectations of each group. Tailoring support structures while preserving cohesion across the float pool team is essential to promoting safety, well‐being and high‐quality care.

### 3.8. Summary

The integration of contextual descriptive and qualitative findings reveals a float pool workforce characterised by versatility, commitment, and adaptive capacity, yet exposed to structural vulnerabilities that leadership practices can directly address.

TL through recognition, developmental support, structured supervision and psychological safety emerges as a key lever for strengthening role clarity, reducing stress and enhancing professional fulfilment within flexible staffing models.

## 4. Discussion

The findings of this study reveal that float pool nursing constitutes a structurally vulnerable model of work embedded in uncertainty, relational discontinuity and constant transitions across organisational microcultures [21]. Participants described entering unfamiliar units where expectations were unspoken, support inconsistent and legitimacy repeatedly renegotiated [20, 21]. These experiences align with the concept of structural vulnerability articulated by Bourgois et al. [3], where institutional configurations differentially expose specific groups to risk. The invisibility of cognitive and relational labour described by participants resonates with Allen’s analysis of the hidden work of registered nurses [4], yet mobility intensifies this invisibility by removing contextual familiarity and undermining recognition [21]. This matters for nursing management because it reframes float pool work as a governance issue rather than a deficit of individual resilience or motivation.

A more balanced reading of these findings also requires recognition of the positive dimensions of float pool work. Participants did not describe mobility only as burden; they also associated the role with dynamism, breadth of experience, adaptability and a sense of organisational usefulness. In this sense, float pool practice may offer opportunities for accelerated learning, broader clinical exposure, and the development of versatile professional competence. However, these positive aspects become sustainable only when mobility is accompanied by role clarity, adequate preparation, supportive reception and consistent organisational recognition.

The recurrent need for rapid adaptation reflects Weick’s theory of sensemaking, whereby professionals operating under ambiguity must rebuild situational meaning to act effectively [37]. Participants described a continuous effort to interpret unit norms, workflows and relational dynamics, often with insufficient information or relational grounding. Such cognitive demands are not merely operational challenges: They undermine the stability required for safe practice and contribute to emotional strain [23, 24, 37].

Psychological safety emerged as a central analytic category. Participants frequently hesitated to ask questions, express uncertainty or seek support when joining new units, behaviours incompatible with safe clinical performance [38, 39]. Edmondson’s foundational work establishes psychological safety as essential for learning, collaboration and error reporting [40]. Findings from Lee and Seo demonstrate that inclusive leadership increases psychological safety and innovative behaviour among registered nurses [41], yet float pool professionals often lack access to consistent leadership relations due to their mobility. The variability in reception across units observed in this study mirrors the organisational antecedents of psychological safety identified by O’Donovan and McAuliffe [39]. Contemporary reviews reinforce that stable relational norms and inclusive climates are necessary for psychological safety in healthcare teams [38, 39]. For mobile staff, psychological safety is not a stable team property but a repeatedly reconstructed condition that depends on predictable reception practices and leadership continuity.

Participants’ accounts also demonstrate significant disruption of professional identity and belonging. Registered nurses described feeling like perpetual outsiders, frequently confronting subtle or explicit signals that questioned their legitimacy [20, 21]. These findings align with research showing that professional identity formation requires stable cultural settings and opportunities for relational integration [42]. Professional values and ethical grounding identified as central components of identity are shaped within consistent team cultures [43], yet float pool registered nurses are deprived of such continuity. Benner’s theory further clarifies this tension: Expert practice is contextually situated and relies on familiarity with unit‐specific patterns [21, 44]. Despite their experience, float pool registered nurses were repeatedly positioned in ‘advanced beginner’ situations due to contextual discontinuity. This identity fragmentation represents both an emotional burden and an organisational inefficiency, as it impedes the full enactment of professional expertise [21, 44].

The emotional strain, vigilance and role ambiguity described by participants in parallel evidence linking inadequate support to burnout and distress in registered nurses [6, 23]. Fatigue‐related risks, including missed care and reduced performance, have been documented among registered nurses working under demanding and unstable conditions [21, 24]. Fischer et al. specifically highlight the resource imbalances and increased job demands in float pool environments [21]. Findings strongly corroborated here. Research on early‐career adaptability similarly shows that insufficient mentoring and lack of relational anchoring hinder professional confidence [25, 45, 46].

Within this context, TL emerged as one of the most significant protective structures identified in the literature and echoed in participants’ narratives. TL has been shown to enhance care performance, promote organisational sustainability, improve well‐being through justice and support and strengthen engagement. Studies also demonstrate positive associations between TL and patient safety behaviours, reduced turnover intention and increased strategic flexibility [10, 14–19]. In other words, TL operates here less as a personal style and more as an organisational mechanism that creates clarity, legitimacy and relational safety under conditions of structural mobility. In this study, the limited moments when float pool registered nurses experienced supportive leadership were described as stabilising and restorative. Conversely, the absence of leadership magnified relational and operational vulnerability. These findings reinforce that TL is essential where mobility, ambiguity and relational discontinuity converge [14, 20, 21, 23].

Another contribution relates to organisational resilience. Participants’ accounts revealed a system where individual adaptability was expected without corresponding structural support [1, 2]. Contemporary resilience literature emphasises that resilience is a multilevel organisational construct, shaped far more by leadership, culture and resources than by personal traits [6, 7]. Evidence shows that resilience influences care quality and turnover intention [8]. Mahdiani and Ungar warn against the ‘dark side’ of resilience, whereby organisations valorise individual endurance while neglecting systemic deficiencies [9]. This pattern was visible in our findings: Float pool registered nurses were celebrated for flexibility, yet lacked the structures needed to perform sustainably. Workforce agility, without formal relational and organisational infrastructures, becomes workforce fragility [1, 2, 9].

From a management perspective, this distinction is crucial: Agility is sustainable only when it is matched by infrastructure for orientation, learning and support.

The study also highlights the importance of clinical supervision and structured support. Participants expressed a need for reflective space, emotional containment, and consistent guidance needs corroborated by evidence demonstrating that clinical supervision improves emotional well‐being and professional development [36, 45, 46]. The professional nurse advocate (PNA) model provides a structured approach to restorative supervision and workforce empowerment [26], and recent scholarship argues for expanded adoption of support structures in nursing and midwifery [27]. However, existing frameworks have not been adapted to the distinctive requirements of mobile roles. These findings suggest that float pool teams require supervisory models specifically tailored to mobility, emphasising relational safety, identity consolidation and structured transition support [21, 42]. A practical implication is that supervision in float pools should be designed as a mobility‐sensitive support structure, not as a generic model imported from static ward teams.

Integrating these findings within broader organisational theory strengthens their interpretive coherence. Schein’s work illustrates how microcultural variation shapes belonging and performance across units [47]. Weick clarifies the cognitive demands imposed by constant reorientation [37]. Edmondson’s theory explains why psychological safety is fragile under mobility [40], while Benner’s analysis highlights the contextual nature of expertise [44]. Together, these perspectives demonstrate that float pool work is not simply an operational mechanism: It is a relational and organisational phenomenon in which culture, leadership and structural conditions intersect, shaping the extent to which mobile registered nurses can practise safely, confidently and with professional integrity [3, 14, 47].

Overall, this study contributes to growing international evidence that flexible staffing systems cannot rely solely on individual adaptability [1, 2, 9]. Float pool registered nurses perform essential organisational work, yet their contributions remain structurally unsupported and relationally undervalued [4, 21]. Taken together, the study specifies a set of actionable organisational conditions through which flexible staffing can be made safer, more learnable and more retainable. Strengthening the sustainability of mobile workforce models requires strategic leadership, coherent cultural practices, formalised induction, tailored supervision and organisational commitment to relational and psychological safety [14, 45–47].

Although the study was conducted in a single large tertiary hospital, the organisational mechanisms identified are likely to be relevant beyond this specific setting. The exact form of float pool work may vary across healthcare systems, staffing models and cultural environments; however, recurrent issues such as uncertain reception, uneven induction, fluctuating legitimacy, role ambiguity and dependence on local leadership are not unique to one institution. The findings are therefore best understood as analytically transferable organisational patterns whose expression will depend on local governance arrangements, unit cultures and leadership infrastructures. While context‐specific, these findings provide transferable insight into how mobile staffing is experienced and governed in high‐complexity hospital systems.

## 5. Implications for Nursing Management

The findings of this study make a substantive contribution to nursing management by showing that float pool nursing is not merely a flexible staffing resource but a complex organisational practice shaped by structural uncertainty, cultural variability and fluctuating relational conditions. These features require management responses that move beyond operational deployment and address the organisational conditions that influence staff experience, performance and safety.

First, the findings support the need to recognise float pool nursing as a specialised area of practice rather than as a residual mechanism for absorbing staffing deficits. Formal role definitions, explicit competency expectations, and structured induction processes are needed to strengthen clarity, legitimacy and consistency across clinical areas. Such measures may also reinforce professional identity and reduce the marginalisation described by participants.

Second, the study identifies leadership as a key organisational condition shaping safety and stability in mobile teams. Leadership is considered here through participants’ accounts of organisational and supervisory practices, with TL used as an interpretive lens rather than as a measured construct. In practice, this points to the importance of visible and relationally attentive leadership behaviours: ensuring consistent induction on arrival, clarifying role boundaries and expectations, identifying an accessible point of contact in receiving units, recognising float pool expertise as legitimate rather than auxiliary, and creating psychologically safe opportunities for questions, feedback, debriefing and reflective support.

Third, nursing managers should implement standardised unit‐level integration processes so that float pool staff are consistently welcomed, orientated and supported at the point of entry. Designated points of contact, brief, structured introductions and explicit communication of expectations can reduce uncertainty and distribute responsibility for relational safety across receiving teams. To be effective, these practices should be organisationally agreed and embedded in routine unit governance rather than left to individual goodwill.

Fourth, the findings highlight the importance of protected reflective and supportive spaces for mobile staff. The emotional and cognitive labour associated with continual adaptation warrants intentional structures for debriefing, mentoring and guided reflection. Such investment may help reduce cumulative strain, support sustainable adaptation and strengthen retention.

Overall, the study suggests that workforce flexibility is sustainable only when matched by parallel investment in organisational infrastructure. Flexibility without structural support produces vulnerability; flexibility supported by leadership, cultural coherence and reflective containment produces adaptive capacity. Table 2 translates the four experiential themes into practical management levers and indicative monitoring markers relevant to safer and more sustainable float pool systems.

**TABLE 2:** Management translation matrix derived from qualitative themes, with contextual checklist descriptors used for practical prioritisation.

Qualitative theme (results)	Organisational risk mechanism	Actionable management response	Implementation notes	Suggested indicators
Theme 1. Polyvalence as identity and structural demand	Role centrality without commensurate recognition; autonomy becomes burdensome under constant reorientation and shifting expectations.	Formalise role recognition and strengthen assignment governance.	Nursing management and unit leaders define expectations, boundaries, deployment criteria, escalation routes and basic welcome practices in receiving units.	Perceived recognition; role clarity; incidents linked to misallocation; satisfaction with assignment decisions.
Theme 2. Uncertainty, stress and formative vulnerability	Safety threats from lack of briefing, high unit‐to‐unit variability and competence gaps during unfamiliar or high‐acuity placements.	Implement a mobility‐specific induction pathway and a unit‐specific first‐hour briefing protocol.	Float pool coordination and receiving charge nurses provide quick unit guidance, competence mapping and updates after protocol changes.	Self‐rated preparedness; stress frequency; near‐miss reporting; time‐to‐orientation; training completion.
Theme 3. Clinical supervision as emotional and developmental scaffolding	Limited supervision leaves emotional strain, uncertainty and learning needs insufficiently contained.	Implement a restorative and developmental clinical supervision pathway tailored to mobile staff.	Nurse managers and trained supervisors provide scheduled supervision, rapid debriefing after high‐impact shifts and clear access routes.	Supervision uptake; well‐being and exhaustion indicators; intention‐to‐leave; perceived support.
Theme 4. Belonging, recognition and the quest for role definition	Psychological safety is weakened when reception is inconsistent, role boundaries are unclear, or delegation is inappropriate.	Introduce psychological safety micro‐practices, explicit role definition and delegation safeguards.	Unit leaders identify a first‐hour reference person, clarify role/task expectations and provide rapid routes for conflict resolution.	Psychological safety; perceived inclusion; role‐boundary incidents; conflict reports; satisfaction with reception.

*Note:* This matrix is an applied translation of the qualitative themes generated from the phenomenological interview sample (*n* = 12; six registered nurses and six nursing assistants). Descriptive contextual checklist patterns from the full float pool workforce (*N* = 22) were used only to support practical prioritisation. They were not treated as quantitative outcome data, inferential evidence or a separate mixed‐methods strand. Suggested indicators are prospective monitoring options and were not measured in this study.

## 6. Conclusion

This study shows that float pool mobility is not a neutral staffing arrangement but an organisational condition that shapes psychological safety, role legitimacy and the enactment of expertise across variable clinical environments. Participants described mobility as work marked by uncertainty, fluctuating legitimacy and uneven support, with consequences not only for emotional well‐being but also for confident practice, collaboration and safe care.

The findings indicate that sustainable mobile workforce models cannot rely on individual adaptability alone. They require organisational arrangements that reduce uncertainty, clarify role boundaries, standardise induction and embed supportive and relational leadership practices across receiving units. Interpreted through a TL lens, the findings suggest that leadership may operate as an organisational condition that either protects or undermines psychological safety and role legitimacy within mobile nursing systems.

By identifying the organisational and relational conditions that shape float pool practice, this study contributes to a more robust understanding of mobile nursing as a specialised and structurally vulnerable form of work. Rather than treating mobility as a stopgap operational response to staffing shortages, the findings support its reconceptualisation as a specialised area of practice requiring intentional design, recognition and sustained managerial support.

The study is limited by its single‐site design and by its focus on float pool staff rather than managerial perspectives. Although meaning‐focused saturation was achieved, the sample cannot capture the full range of mobile nursing experiences across settings. Future research should extend this work through multisite and comparative designs, and by examining induction, psychological safety, supervision and governance mechanisms that support sustainable mobile nursing practice.

## Author Contributions

Rubén Llada Suarez: conceptualisation, methodology, investigation, formal analysis, writing–original draft and writing–review and editing. Maria Antuna‐Casal: conceptualisation, methodology, supervision, formal analysis, project administration, writing–original draft, writing–review and editing and corresponding author. Lucía del Fresno Marqués: methodology, formal analysis, validation and writing–review and editing. Marta Manuz Pérez, Yolanda García Rodríguez, Margarita González Pérez and Carmen Álvarez Fanjul: investigation, resources, contextual expertise, validation and writing–review and editing.

## Funding

This research received no specific grant from any funding agency in the public, commercial or not‐for‐profit sectors.

## Disclosure

No funder had any role in the design of the study, data collection, analysis, interpretation, manuscript preparation, or the decision to publish. The authors reviewed and approved the final manuscript, agreed to be accountable for all aspects of the work and take full responsibility for its accuracy and integrity. The study was conducted as part of the authors’ academic and professional roles.

## Ethics Statement

This study was conducted in accordance with the ethical principles of the Declaration of Helsinki and received formal approval from the Ethics Committee for Research with Medicines of the Principality of Asturias (CEImPA 2025.082). Institutional authorisation was also granted by the participating hospital. All participants received written and verbal information about the study aims, procedures, confidentiality safeguards, data handling and their right to withdraw at any time without consequences. Written informed consent was obtained from all participants prior to data collection. Interviews were conducted in private settings within the hospital. Audio‐recordings, verbatim transcripts and analytic files were anonymised and stored on secure, password‐protected institutional systems in accordance with applicable data protection regulations.

## Conflicts of Interest

The authors declare no conflicts of interest.

## Supporting Information

Additional supporting information can be found online in the Supporting Information section.

## Supporting information


**Supporting Information 1** Supporting File S1. Interview guide. The semistructured interview schedule used for individual interviews with float pool registered nurses and nursing assistants (eight open‐ended questions and prompts).

**Supporting Information 2** Supporting File S2. SRQR and COREQ reporting checklist (abridged narrative). A consolidated narrative checklist describing how the study adheres to SRQR and COREQ reporting standards, to support transparency and appraisal of methodological rigour.

**Supporting Information 3** Supporting File S3. Coding framework/codebook (abridged). The analytic tree aligned with the four experiential themes reported in the Results, including operational definitions for codes and brief coding illustrations to support auditability.

**Supporting Information 4** Supporting File S4. Audit trail excerpt. An excerpt of the audit trail documenting key analytic decisions and iterative steps undertaken during coding, theme development and verification.

**Supporting Information 5** Supporting File S5. Management translation matrix. A practice‐facing matrix translating the four experiential themes into actionable managerial levers and monitoring indicators relevant to safe and sustainable float pool implementation.

**Supporting Information 6** Supporting File S6. Contextual checklist distributions. Descriptive distributions from the preparatory contextual checklist (contextual dataset) reporting perceived stressors, preparedness and priority training needs among the float pool team.

## Data Availability

Due to the sensitive nature of the qualitative interview data and the risk of deductive identification within a small professional group, the full interview transcripts are not publicly available. De‐identified excerpts supporting the findings may be made available from the corresponding author upon reasonable request, subject to ethical approval and institutional restrictions.
