# Supplementary material for: Organisational and Governance Conditions Shaping Psychological Safety and Structural Vulnerability in Float Pool Nursing: A Qualitative Study
Source: J Nurs Manag. 2026 Jul 19;2026:1427120. doi: 10.1155/jonm/1427120 (PMC13382358; doi:10.1155/jonm/1427120)
Supplement: Supplementary file 1 — Supporting Information 1 Supporting File S1. Interview guide. The semistructured interview schedule used for individual interviews with float pool registered nurses and nursing assistants (eight open‐ended questions and prompts). [file JONM-2026-1427120-s006.docx]

***Supplementary File S1. Interview guide.***

*The semi-structured interview schedule used for individual interviews with float pool registered nurses and nursing assistants (eight open-ended questions and prompts).*

| *Item- Questions* | *Text* |
| --- | --- |
| *Q1 (Spanish, from transcript)* | *¿Qué significado tiene para ti el rol del retén de enfermería en el Hospital?* |
| *Q1 (English, author translation)* | *What does the float pool nursing role mean to you within this hospital??* |
| *Q2 (Spanish, from transcript)* | *¿En qué unidades satélites de las que estás destinada te sientes más cómoda, trabajando como retén y por qué te sientes más cómoda, en esos sitios?* |
| *Q2 (English, author translation)* | *In which satellite unit do you feel most comfortable working as float pool staff, and why?* |
| *Q3 (Spanish, from transcript)* | *¿Cuáles son las situaciones o factores en tu trabajo que crees que más contribuyen al estrés al agotamiento físico y emocional y si puedes contarme alguna experiencia tuya?* |
| *Q3 (English, author translation)* | *Which situations or factors at work contribute most to your stress and physical exhaustion? Can you tell me about a particular experience?* |
| *Q4 (Spanish, from transcript)* | *¿Existen situaciones específicas en las que sientas que no posees los conocimientos y habilidades específicas necesarias para el puesto, y si hay alguno de ellos, en qué situaciones? ¿Qué situaciones son?* |
| *Q4 (English, author translation)* | *Are there specific situations in which you feel you do not have the knowledge and/or the specific skills required for the position? Which situations are they?* |
| *Q5 (Spanish, from transcript)* | *¿Qué tipo de formación opinas tu que sería más beneficiosa para ti, concretamente en tu rol actual, cuando vas cambiando de sitio, cuando vienes a trabajar?* |
| *Q5 (English, author translation)* | *What type of training do you consider would be most beneficial for you in your current role, and why?* |
| *Q6 (Spanish, from transcript)* | *¿Qué entiendes por supervisión clínica y de qué manera crees que podría influir en tu desempeño y bienestar en el trabajo?* |
| *Q6 (English, author translation)* | *What do you understand by clinical supervision, and how do you think it could influence your performance and wellbeing at work?* |
| *Q7 (Spanish, from transcript)* | *¿Qué temas crees que deberían ser prioritarios en un programa de supervisión clínica y por qué son importantes para ti esos temas?* |
| *Q7 (English, author translation)* | *From your perspective, what topics should be priorities in a clinical supervision programme? Why are they important to you?* |
| *Q8 (Spanish, from transcript)* | *¿Hay algo más que te gustaría compartir sobre tu experiencia como retén de enfermería?* |
| *Q8 (English, author translation)* | *Is there anything else you would like to share about your experience as float pool (retén) staff?* |
