# Supplementary material for: Organisational and Governance Conditions Shaping Psychological Safety and Structural Vulnerability in Float Pool Nursing: A Qualitative Study
Source: J Nurs Manag. 2026 Jul 19;2026:1427120. doi: 10.1155/jonm/1427120 (PMC13382358; doi:10.1155/jonm/1427120)
Supplement: Supplementary file 2 — Supporting Information 2 Supporting File S2. SRQR and COREQ reporting checklist (abridged narrative). A consolidated narrative checklist describing how the study adheres to SRQR and COREQ reporting standards, to support transparency and appraisal of methodological rigour. [file JONM-2026-1427120-s004.docx]

***Supplementary File S2.* *SRQR and COREQ reporting checklist (abridged narrative).***

A consolidated narrative checklist describing how the study adheres to SRQR and COREQ reporting standards, to support transparency and appraisal of methodological rigour.

**Integrated SRQR and COREQ Narrative Checklist**

This supplementary file provides a consolidated narrative account of how the study adhered to established qualitative reporting standards, integrating the Standards for Reporting Qualitative Research (SRQR) and the Consolidated Criteria for Reporting Qualitative Research (COREQ). In accordance with Wiley journal guidance, the checklist is presented in descriptive narrative form to support transparency, methodological integrity and appraisal of rigour.

**A. COREQ (Abbreviated Narrative Reporting)**

**Research team and reflexivity**

Interviews were conducted by members of the hospital on-call coordination team who had received formal training in qualitative research methods, including in-depth interviewing and qualitative inquiry in health sciences. Interviewers occupied coordination and supervisory roles within the organisation but were not members of the float pool team, enabling an appropriate balance between contextual understanding and analytic distance.

Researcher positioning and reflexive considerations are described in the Methods section of the main manuscript. Reflexive awareness was maintained throughout the study regarding professional proximity to the setting and its potential influence on data generation and interpretation.

**Participant identification and coding**

To ensure confidentiality and analytic traceability, each participant was assigned an alphanumeric code reflecting professional role and interview sequence. Registered nurses were coded as RN1–RN6 and nursing assistants as NA1–NA6. These codes were used consistently across transcripts, analytic files, coding matrices and illustrative quotations presented in the Results section.

The correspondence between participant identities and codes was stored separately from the research dataset and was accessible only to the principal investigator for data protection and audit purposes. No personal identifiers were included in transcripts, qualitative software, or reporting materials.

**Study design**

A qualitative descriptive phenomenological design was employed, grounded in Husserlian philosophy and operationalised through Colaizzi’s analytic method. This design was selected to explore the essence of lived experience within a professional role characterised by high mobility, uncertainty and relational discontinuity. The methodological rationale is fully detailed in the Study design subsection of the Methods.

**Sampling and participants**

Purposive sampling was used to recruit participants from the hospital float pool team. Twelve participants (six registered nurses and six nursing assistants) were selected on the basis of active participation in the float pool model, stability in the role, sufficient length of experience to support reflective articulation, and variation in professional role and cross-unit practice, in order to obtain a sufficiently diverse and information-rich account of the phenomenon under study.

Participation was voluntary and based on written informed consent. All individuals invited to participate agreed to do so; no refusals or withdrawals occurred. Although the sample size was defined through purposive selection rather than iterative expansion, ongoing analysis confirmed that conceptual saturation was reached, defined as the point at which no new experiential meanings or thematic variations emerged.

**Setting**

The study was conducted in a large tertiary hospital operating a hospital-wide float pool system. Interviews took place in a private workroom within the hospital to ensure confidentiality and minimise interruptions. To preserve institutional anonymity, contextual description was intentionally non-specific.

**Data collection**

Data were collected through individual semi-structured interviews, guided by an interview schedule comprising eight open-ended questions exploring role meaning, comfort across units, stressors, perceived competence, training needs and experiences of clinical supervision. The interview guide is presented in Supplementary File S1 and summarised in Figure 1 of the main manuscript.

Interviews lasted approximately 30–60 minutes. All interviews were audio-recorded with participant consent and transcribed verbatim.


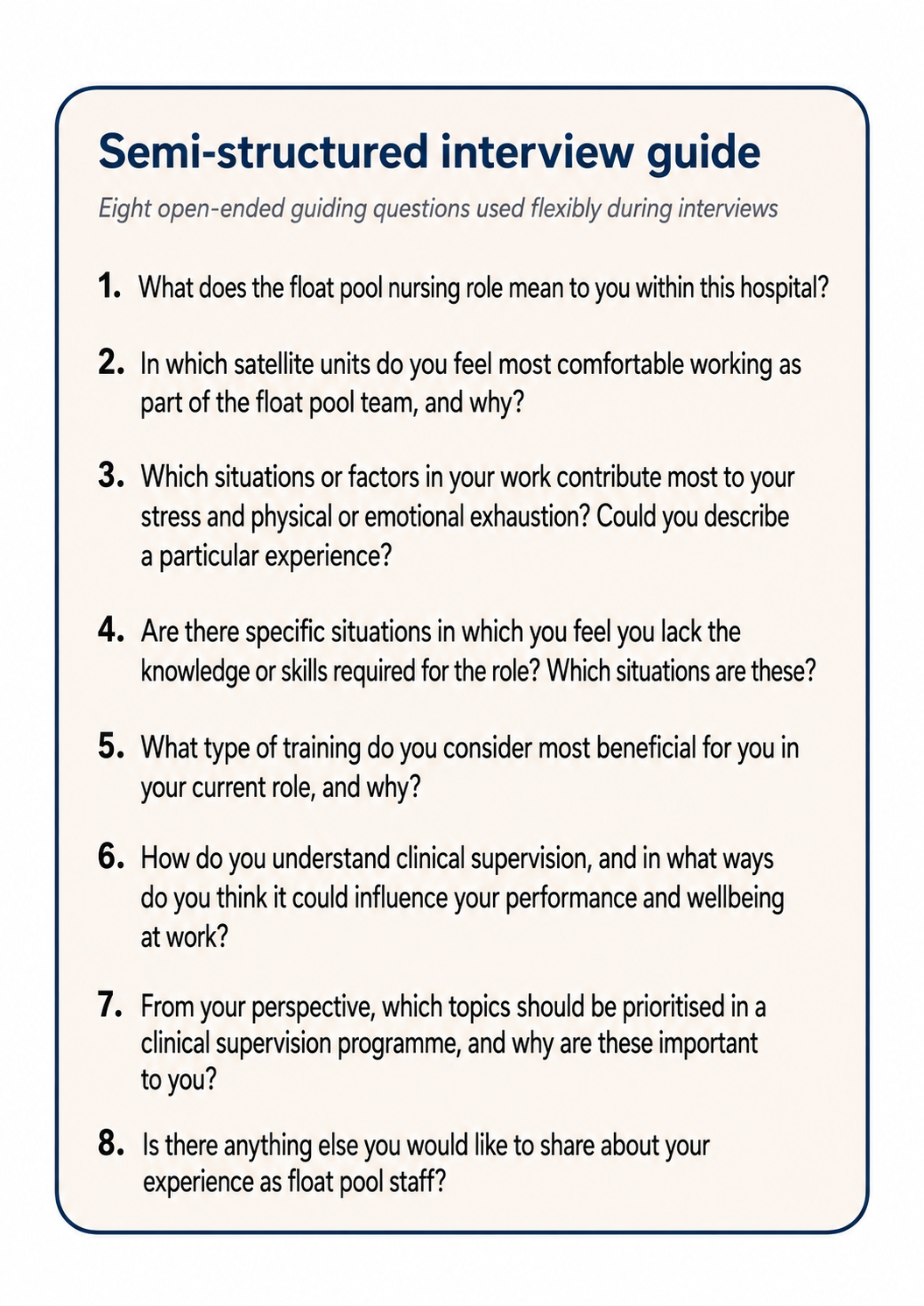


***Figure 1. Semi-structured interview guide used for data collection.***

Note. The interview guide comprised eight open-ended guiding questions used flexibly during the interviews. These questions were intended to support conversational coverage of role meaning, adaptation across units, stressors, perceived competence, training needs, clinical supervision, and additional experiences. They were not treated as pre-specified analytic categories. The qualitative findings are reported separately as themes generated through the phenomenological analysis.

Data collection took place between March and May 2025. Interviews were conducted in Spanish; quotations reproduced in English in the manuscript were translated by the authors for reporting purposes. No follow-up interviews were conducted.

Field notes were taken during and immediately after each interview to capture contextual information, pauses, interruptions (e.g. pager alerts), non-verbal cues and the emotional tone of interactions. These notes informed analytic reflexivity but were not treated as standalone data.

**Participant checking**

Transcripts and themes were **not returned to participants** for member checking. This decision was taken to minimise risks of re-identification within a small and highly recognisable professional group. All data were securely stored and analysed in anonymised form.

**Data analysis**

Analysis followed Colaizzi’s phenomenological method, progressing through immersive reading, identification of meaning units, open coding, axial clustering and thematic synthesis. Coding was iterative and reflexive, combining descriptive fidelity with progressive abstraction.

Data management and coding were supported by OpenCode version 4.3 (Umeå University). Peer discussion among members of the research team was used to refine coding decisions and resolve interpretive discrepancies, thereby strengthening analytic credibility.

**Reporting**

Findings are illustrated using verbatim quotations attributed to participant codes (RN1–RN6; NA1–NA6). Themes are presented with sufficient contextual and interpretive depth to enable readers to assess coherence with the data and potential transferability.

**B. SRQR (Abbreviated Narrative Reporting)**

**Qualitative approach and research paradigm**

The study adopted a descriptive phenomenological approach to examine how float pool registered nurses and nursing assistants experience their role under conditions of organisational mobility and uncertainty.

**Context and sampling strategy**

The organisational context, inclusion criteria and purposive sampling strategy are described in the Methods section of the main manuscript. Saturation tracking ensured sufficient depth and thematic coherence of experiential data.

**Ethical considerations**

The study received approval from the Ethics Committee for Research with Medicines of the Principality of Asturias (CEImPA 2025.082) and institutional authorisation from the participating hospital. Written informed consent was obtained from all participants. Confidentiality and data protection were rigorously maintained throughout the research process.

**Data collection methods**

Data collection combined a brief author-developed contextual checklist with in-depth semi-structured interviews. The contextual checklist informed refinement of the interview guide and contextual understanding but was not used for statistical inference.

**Data analysis and rigour**

Analytic procedures are transparently described, including coding strategy, use of qualitative software and reflexive practices. Trustworthiness was enhanced through triangulation, audit trail documentation, peer discussion and reflexive journaling. Limitations related to transferability and interpretive scope are addressed in the Discussion.

**Integration with empirical data**

All analytic claims are grounded in participant narratives and supported by illustrative quotations presented in the Results section.

**Reporting guidelines cited**

• **SRQR**: O’Brien, B. C., Harris, I. B., Beckman, T. J., Reed, D. A., & Cook, D. A. (2014). *Standards for reporting qualitative research: A synthesis of recommendations*. Academic Medicine, 89(9), 1245–1251.

• **COREQ**: Tong, A., Sainsbury, P., & Craig, J. (2007). *Consolidated criteria for reporting qualitative research (COREQ): A 32-item checklist for interviews and focus groups*. International Journal for Quality in Health Care, 19(6), 349–357.
