# Supplementary material for: Organisational and Governance Conditions Shaping Psychological Safety and Structural Vulnerability in Float Pool Nursing: A Qualitative Study
Source: J Nurs Manag. 2026 Jul 19;2026:1427120. doi: 10.1155/jonm/1427120 (PMC13382358; doi:10.1155/jonm/1427120)
Supplement: Supplementary file 3 — Supporting Information 3 Supporting File S3. Coding framework/codebook (abridged). The analytic tree aligned with the four experiential themes reported in the Results, including operational definitions for codes and brief coding illustrations to support auditability. [file JONM-2026-1427120-s003.docx]

***Supplementary File S3.* *Coding framework / codebook (abridged).***

The analytic tree aligned with the four experiential themes reported in the Results, including operational definitions for codes and brief coding illustrations to support auditability.

This file provides a concise, auditable description of the coding framework used for the qualitative interview analysis. The analytic tree is aligned with the four experiential themes reported in the Results section, followed by an abridged codebook with operational definitions and two brief coding illustrations.

# A. Analytic tree (themes and associated codes)

**Theme 1. Polyvalence as identity and structural demand**

Associated codes: Polyvalence / functional versatility; Adaptability; Operational reinforcement; Collaborative support; Familiarity with unit environment and equipment; Sense of responsibility; Unrealistic expectations

**Theme 2. Uncertainty, stress and formative vulnerability**

Associated codes: Operational uncertainty; Communication gaps / lack of information; Workload overload; Resource scarcity; Training insecurity / need for refreshers; Formative vulnerability; Self-directed training / self-learning; Guided rotations (practice-based preparation); Theoretical updates / briefings; Structured training

**Theme 3. Clinical supervision as emotional and developmental scaffolding**

Associated codes: Clinical supervision / designated mentor (reference person); Restorative supervision; Formal induction / welcome structure; Workplace mediation; Team cohesion

**Theme 4. Belonging, recognition and the quest for role definition**

Associated codes: Sense of belonging; Welcoming climate; Invisibility; Limited recognition; Professional legitimation; Role/task definition

# B. Abridged codebook (operational definitions)

| Theme (Results) | | Code | | Operational definition |
| --- | --- | --- | --- | --- |
| Theme 1. Polyvalence as identity and structural demand | Polyvalence / functional versatility | | Defining the float pool role as a ‘nurse for everything’; ability to cover any unit. | |
|  | Adaptability | | Capacity to adapt to different units, routines, and microcultures. | |
|  | Operational reinforcement | | Being deployed to reinforce staffing during workload peaks or operational disruption. | |
|  | Collaborative support | | Providing direct support to unit colleagues as routine reinforcement. | |
|  | Familiarity with unit environment and equipment | | Efficiency derived from previous exposure to units, layouts, and equipment. | |
|  | Sense of responsibility | | Perceived duty to cover needs; expectation to ‘know a bit of everything’. | |
|  | Unrealistic expectations | | Assumptions that float pool staff should function as permanent unit staff without induction. | |
| Theme 2. Uncertainty, stress and formative vulnerability | Operational uncertainty | | Arriving without anticipatory information; lack of handover/briefing. | |
|  | Communication gaps / lack of information | | Absence of updates, changes, or clear unit-specific guidance/protocols. | |
|  | Workload overload | | High patient volume and/or inadequate staffing ratios. | |
|  | Resource scarcity | | Insufficient staff, equipment, or materials. | |
|  | Training insecurity / need for refreshers | | Perceived skill gaps; need for updates or refreshers for unit-specific demands. | |
|  | Formative vulnerability | | Role vulnerability arising from mobility without structured preparation and support. | |
|  | Self-directed training / self-learning | | Learning independently; ‘having to figure it out’ while on shift. | |
|  | Guided rotations (practice-based preparation) | | Proposal of structured rotations across services to consolidate competence. | |
|  | Theoretical updates / briefings | | Request for short, targeted theoretical updates. | |
|  | Structured training | | Need for unit-specific orientation materials and written protocols. | |
| Theme 3. Clinical supervision as emotional and developmental scaffolding | Clinical supervision / designated mentor (reference person) | | Supervision understood as having a consistent reference person for guidance. | |
|  | Restorative supervision | | Supervision that includes emotional containment and reflective processing. | |
|  | Formal induction / welcome structure | | Request for explicit induction plans and welcome processes. | |
|  | Workplace mediation | | Access to mediation or supervisory intervention for conflict and tension. | |
|  | Team cohesion | | Peer support and cohesion as a protective buffer. | |
| Theme 4. Belonging, recognition and the quest for role definition | Sense of belonging | | Feeling accepted and valued; pride in the float pool identity. | |
|  | Welcoming climate | | Importance of how receiving units welcome and position float pool staff. | |
|  | Invisibility | | Perceived lack of institutional recognition and visibility. | |
|  | Limited recognition | | Mixed experiences of appreciation, often paired with overload. | |
|  | Professional legitimation | | Training and supervision experienced as legitimising and stabilising the role. | |
|  | Role/task definition | | Need for explicit role descriptions and boundaries (including nursing assistants). | |

# C. Brief coding illustrations (2 examples)

**Example 1. Polyvalence as identity**

Excerpt (anonymised): “For me, being float pool nursing staff means being a ‘nurse for everything’.” (RN1)

| Assigned code(s) | Rationale |
| --- | --- |
| Polyvalence / functional versatility | Defines the role in terms of transversal versatility (‘for everything’) as a core identity marker. |
| Unrealistic expectations (if co-present in context) | May co-occur when polyvalence is framed as an obligation to assume any task without induction or limits. |

**Example 2. Uncertainty on arrival**

Excerpt (anonymised): “When you arrive… no one tells you anything; you feel completely disoriented at first, trying to work out what is needed …” (NA1)

| Assigned code(s) | Rationale |
| --- | --- |
| Operational uncertainty | Absence of anticipatory information/handover increases situational reorientation demands. |
| Communication gaps / lack of information (if confirmed in full excerpt) | Co-occurs when uncertainty is explicitly attributed to missing updates, unclear guidance, or lack of a reference person. |

Note: excerpts are presented in shortened, anonymised form; coding was conducted on full verbatim transcripts.
