# Supplementary material for: Organisational and Governance Conditions Shaping Psychological Safety and Structural Vulnerability in Float Pool Nursing: A Qualitative Study
Source: J Nurs Manag. 2026 Jul 19;2026:1427120. doi: 10.1155/jonm/1427120 (PMC13382358; doi:10.1155/jonm/1427120)
Supplement: Supplementary file 4 — Supporting Information 4 Supporting File S4. Audit trail excerpt. An excerpt of the audit trail documenting key analytic decisions and iterative steps undertaken during coding, theme development and verification. [file JONM-2026-1427120-s002.docx]

***Supplementary File S4. Audit trail excerpt.***

An excerpt of the audit trail documenting key analytic decisions and iterative steps undertaken during coding, theme development and verification.

This file provides an audit trail excerpt documenting analytic traceability (data, codes, themes, management translation) and key analytic decisions. It is designed to support transparency and reproducibility, consistent with qualitative reporting expectations.

# A. Traceability map (data-findings-translation)

| Input | Output | How it is documented |
| --- | --- | --- |
| Verbatim transcripts (audio → transcription) | Meaning units | Participant-coded quotations (RN1–RN6 / NA1–NA6) used for traceable excerpts. |
| Meaning units | Initial codes | Descriptive and analytic coding captured in the abridged codebook (Supporting Information S3). |
| Codes | Results | Iterative grouping into the four experiential themes reported in Results. |
| Results | Management translation (Table 2; expanded in S5) | Linking themes to risk mechanisms and implementable actions. |

# B. Decision log (abridged)

| Stage | Decision | Rationale | Where evidenced |
| --- | --- | --- | --- |
| Study framing | Contextual checklist treated as preparatory orientation rather than a mixed-methods design. | Avoids methodological inconsistency; qualitative interpretation remains phenomenological. | Methods and Results (contextual checklist + S6). |
| Interview design | Semi-structured guide with eight core questions. | Ensures comparability while enabling depth and probes. | Figure 1 and Supporting Information S1. |
| Data handling | Verbatim transcription + anonymisation with RN1–RN6 / NA1–NA6 codes. | Protects confidentiality while preserving quote-level traceability. | Methods; Supporting Information S3 (examples). |
| Coding approach | Iterative descriptive + analytic coding; refinement through consolidation. | Captures manifest content and meaning structures; supports progressive abstraction. | Methods; Supporting Information S3. |
| Software | OpenCode 4.3 used for coding management. | Supports auditable code/category history. | Methods. |
| Synthesis | Codes consolidated into four experiential themes (Results). | Moves from codes to coherent organisational patterns while retaining anchoring. | Results; Supporting Information S3. |
| Trustworthiness | Peer review/triangulation with consensus resolution. | Reduces interpretive bias and strengthens credibility. | Methods (trustworthiness). |
| Translation | Themes translated into management actions via Table 2; expanded matrix in S5. | Aligns findings with Journal of Nursing Management’s applied orientation. | Table 2; Supporting Information S5. |

# C. Structured analytic memo (excerpt)

**Interpretive focus**

Mobility is an organisational condition that imposes sustained cognitive and relational demands: continuous reorientation, negotiation of belonging, and exposure to heterogeneous unit expectations.

**Empirical cues**

Participants describe the role as ‘for everything’, coupled with accounts of uncertainty on arrival, variable reception, and formative vulnerability when high-acuity placements occur without structured preparation.

**Organisational reading**

Strain is not reducible to workload volume: it is shaped by structural conditions (absence of systematic induction, microcultural variability, communication deficits) and limited access to restorative supervision.

**Implications**

Priority interventions include mobility-specific induction and briefing protocols; guided rotations and competence mapping; restorative supervision; role definition and delegation safeguards; and escalation/mediation pathways.
