# Supplementary material for: Organisational and Governance Conditions Shaping Psychological Safety and Structural Vulnerability in Float Pool Nursing: A Qualitative Study
Source: J Nurs Manag. 2026 Jul 19;2026:1427120. doi: 10.1155/jonm/1427120 (PMC13382358; doi:10.1155/jonm/1427120)
Supplement: Supplementary file 5 — Supporting Information 5 Supporting File S5. Management translation matrix. A practice‐facing matrix translating the four experiential themes into actionable managerial levers and monitoring indicators relevant to safe and sustainable float pool implementation. [file JONM-2026-1427120-s001.docx]

***Supplementary File S5. Management translation matrix.***

This supplementary matrix provides a practice-facing translation of the four qualitative themes into actionable managerial levers, implementation considerations, and suggested monitoring indicators relevant to safe and sustainable float pool implementation. It follows the same column structure as Table 2 in the manuscript and provides additional implementation detail to enhance practical utility. The matrix is an applied synthesis of qualitative findings rather than primary data. Contextual checklist descriptors from the full float pool workforce (N = 22) were used only to support practical prioritisation after theme generation. They were not used to generate themes, test associations, support statistical inference, or constitute a separate mixed-methods component.

| Qualitative theme (Results) | Organisational risk mechanism | Actionable management response | Implementation notes (who/what) | Suggested indicators |
| --- | --- | --- | --- | --- |
| Theme 1. Polyvalence as identity and structural demand | “Essential but invisible”: role centrality without commensurate recognition; autonomy becomes burdensome under constant reorientation and shifting expectations. | Formalise role recognition and strengthen assignment governance (define expectations, boundaries, and criteria for deployment). | Nursing management + unit leaders: publish a brief float-pool role charter; define escalation routes for unsafe/inappropriate assignments; introduce a simple welcome micro-practice in receiving units (who-to-call, where-to-find essentials, what-changes-today). | Perceived recognition; role-clarity ratings; incidents linked to misallocation; satisfaction with assignment decisions; reported ‘invisibility’ narratives over time. |
| Theme 2. Uncertainty, stress and formative vulnerability | Safety threats from lack of briefing/handover, high unit-to-unit variability, and competence gaps during high-acuity placements. | Implement a mobility-specific induction pathway plus a unit-specific “first-hour” briefing protocol. | Float pool coordination + receiving charge nurse: standardised first-hour briefing checklist; accessible unit quick-guides; competence map to match assignments; structured ‘update’ huddles after major protocol changes. | Self-rated preparedness; stress frequency; near-miss reporting; time-to-orientation; documented competency gaps and training completion rates. |
| Theme 3. Clinical supervision as emotional and developmental scaffolding | Low availability/uptake of supervision; unmet need for containment, reflective processing, and feedback in a role characterised by repeated transitions. | Implement a restorative/clinical supervision pathway tailored to mobility. | Nurse manager + trained supervisors: scheduled supervision cycles (e.g., monthly/bi-monthly); rapid debrief after high-impact shifts; clear access routes, confidentiality parameters, and referral thresholds. | Supervision uptake; wellbeing/exhaustion indicators; intention-to-leave; moral distress reports; perceived support scores. |
| Theme 4. Belonging, recognition and the quest for role definition | Psychological safety is fragile when reception is inconsistent; boundary violations and inappropriate delegation increase relational strain and perceived risk. | Introduce psychological safety micro-practices plus explicit role-definition and delegation safeguards. | Unit leaders: explicit task allocation norms; named first-hour reference person for orientation; rapid conflict-resolution/mediation pathway; role/task clarity brief for nursing assistants to reduce inappropriate delegation. | Psychological safety ratings; conflict-report frequency; reports of inappropriate delegation; belonging/acceptance ratings; complaints/escalations related to role ambiguity. |
