# Supplementary material for: Organisational and Governance Conditions Shaping Psychological Safety and Structural Vulnerability in Float Pool Nursing: A Qualitative Study
Source: J Nurs Manag. 2026 Jul 19;2026:1427120. doi: 10.1155/jonm/1427120 (PMC13382358; doi:10.1155/jonm/1427120)
Supplement: Supplementary file 6 — Supporting Information 6 Supporting File S6. Contextual checklist distributions. Descriptive distributions from the preparatory contextual checklist (contextual dataset) reporting perceived stressors, preparedness and priority training needs among the float pool team. [file JONM-2026-1427120-s005.docx]

***Supplementary File S6. Contextual checklist distributions***

Descriptive distributions from the preparatory contextual checklist (contextual dataset) reporting perceived stressors, preparedness, and priority training needs among the float pool team.

This supplement provides descriptive distributions from the preparatory contextual checklist completed by the wider float pool workforce during the study period (N = 22). The phenomenological interview sample comprised twelve purposively selected key informants (n = 12; six registered nurses and six nursing assistants). The contextual checklist was used to orient the qualitative phase, refine the interview guide, and support practical prioritisation; it was not used as a separate quantitative analytic strand.

**Table S6_1. Participant characteristics (contextual checklist)**

| Variable | Registered nurses | Nursing Assistants | Total |
| --- | --- | --- | --- |
| Sex | Predominantly female | Predominantly female | — |
| Years in float pool role | All >6 years | Majority >6 years | 75% >6 years |
| Employment status | Active float pool role | Active float pool role | 100% |
| Clinical mobility level | High | High | — |
| Prior mobility training | Informal / limited | Informal / limited | 100% unstructured |

**Table S6_2. Contextual checklist stressors and Exhaustion levels**

| Contextual area | Variable | % Reporting |
| --- | --- | --- |
| Organisational stressors | Workload overload | 75% |
| Organisational stressors | Difficult clinical situations | 63% |
| Organisational stressors | Insufficient staffing | 54% |
| Structural stressors | Continuous rotation between units | 50% |
| Structural stressors | Lack of supervision | 8% |
| Perceived stress levels | Occasional stress | 67% |
| Perceived stress levels | Frequent stress | 25% |
| Exhaustion levels | Moderate exhaustion | 63% |
| Exhaustion levels | High exhaustion | 4% |

**Table S6_3. Preparedness, training needs, and clinical supervision (contextual checklist)**

| Contextual area | Item | % Reporting |
| --- | --- | --- |
| Preparedness | Felt fully prepared for float pool assignments | 21% |
| Preparedness | Reported competency gaps | 79% |
| Training need | Identified need for specific technical training | 83% |
| Clinical supervision | Reported knowing about clinical supervision | 29% |
| Clinical supervision | Had never received clinical supervision | 58% |
| Clinical supervision | Had experienced clinical supervision frequently | 4% |
| Perceived value | Believed supervision would enhance their potential | 75% |
| Engagement | Willingness to participate in an innovative supervisory programme | 71% |

**Table S6_4. Self-reported competence across clinical areas**

| Clinical Area | Registered nurses (%) | Nursing Assistants (%) | Least Prepared Group |
| --- | --- | --- | --- |
| Outpatient Parenteral Antimicrobial Therapy | 100 | — | Registered nurses |
| Endoscopy | 91 | 46 | Nursing Assistants |
| Emergency Department | 64 | — | Nursing Assistants |
| PACU / Recovery | 55 | — | Nursing Assistants |
| Paediatrics | — | 77 | Registered nurses |
| Labour Ward | — | 70 | Registered nurses |
| Oncology | 36 | — | Registered nurses |
| Critical Care | 9 | 46 | Both |
| Central Sterile Services Department | — | 38 | Nursing Assistants |
| Operating Theatre | — | 38 | Nursing Assistants |

**Table S6_5. Priority topics for a clinical supervision programme**

| Contextual area | Specific Focus | Priority Level |
| --- | --- | --- |
| Emotional support | Psychological containment; safe relational space | Very high |
| Interpersonal processes | Communication; conflict resolution; team dynamics | High |
| Structured induction | Orientation; written protocols; role clarity | High |
| Technical performance | Ongoing updates; advanced skills | High |
| Reflective practice | Guided reflection sessions | Moderate–High |
| Decision-making support | Guidance for complex unfamiliar scenarios | Moderate |
| Workplace climate | Mediation; psychological safety | Moderate–High |


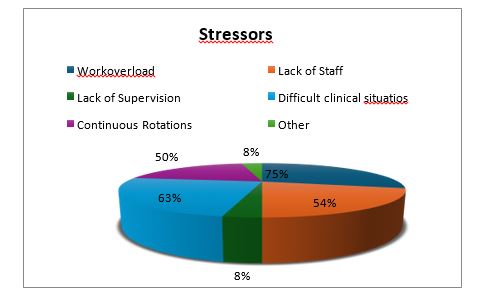


***Figure S6-1. Contextual checklist stressor frequencies Among Float Pool Staff***


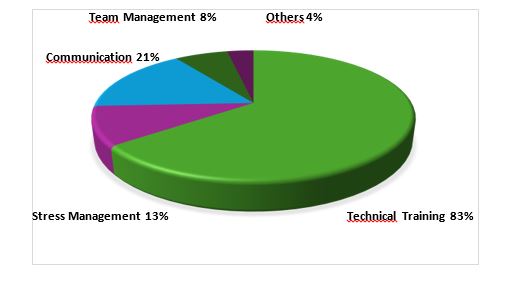


***Figure S6-2. Perceived preparedness and priority training needs among float pool staff***
